# Interplay between medical and interventional therapies in valvular heart disease and heart failure: an expert opinion paper

**DOI:** 10.1093/eschf/xvaf027

**Published:** 2026-01-08

**Authors:** Matteo Pagnesi, Mauro Riccardi, Francesco Maisano, Elena-Laura Antohi, Vassilis Barberis, Magdy Abdelhamid, Henrike Arfsten, Julia Grapsa, Nicole Karam, Denisa Muraru, Karl-Philipp Rommel, Anna Sannino, Wilfried Mullens, Marianna Adamo, Marco Metra

**Affiliations:** Department of Medical and Surgical Specialties, Institute of Cardiology, ASST Spedali Civili, Radiological Sciences, and Public Health, University of Brescia, Brescia, Italy; Institute of Cardiology, ASST Cremona, Cremona, Italy; Department of Cardiac Surgery, San Raffaele Scientific Institute, Milan, Italy; Vita-Salute San Raffaele University, Milan, Italy; Department of Cardiology, Emergency Institute for Cardiovascular Diseases ‘Prof. C.C. Iliescu’, Bucharest, Romania; American Medical Center, American Heart Institute, Nicosia, Cyprus; Faculty of Medicine, Department of Cardiovascular Medicine, Kasr Al Ainy, Cairo University, Giza, Egypt; Division of Cardiology, Department of Internal Medicine II, Medical University of Vienna, Vienna, Austria; Heart and Vascular Institute, Brigham and Women’s Hospital, Harvard Medical School, Boston, MA, USA; Cardiology Department, Clemenceau Medical Center, Balamand University, Beirut, Lebanon; Department of Medicine and Surgery, University of Milano-Bicocca, Milan, Italy; Department of Cardiology, Istituto Auxologico Italiano, IRCCS, Milan, Italy; Department of Cardiology, University Medical Center of the Johannes Gutenberg University, Mainz, Germany; DZHK, German Center for Cardiovascular Research, Partner Site Rhein/Main, Mainz, Germany; Department of Cardiology, Deutsches Herzzentrum der Charité, Berlin, Germany; Friede Springer-Cardiovascular Prevention Center, Charité Universitaetsmedizin Berlin, Berlin, Germany; Department of Cardiology, Ziekenhuis Oost-Limburg, Genk, Belgium; UHasselt, Biomedical Research Institute, Faculty of Medicine and Life Sciences, LCRC, Diepenbeek, Belgium; Department of Medical and Surgical Specialties, Institute of Cardiology, ASST Spedali Civili, Radiological Sciences, and Public Health, University of Brescia, Brescia, Italy; Vita-Salute San Raffaele University, Milan, Italy; Department of Cardiology, Vita-Salute San Raffaele University and IRCCS San Raffaele Hospital, Milan, Italy

**Keywords:** Heart failure, Valvular heart disease, Mitral regurgitation, Aortic stenosis, Aortic regurgitation, Tricuspid regurgitation

## Abstract

Heart failure (HF) and valvular heart disease (VHD) often coexist and share complex pathophysiological pathways. Traditional management strategies follow a step-by-step approach, prioritizing guideline-directed medical therapy (GDMT) and reserving interventional (surgical or percutaneous) options only in case of persistent symptoms or worsening HF after GDMT. However, greater experience with the use of percutaneous procedures, even in high-risk patients, could support a more integrated approach, which exploits the synergistic effects of medical and interventional therapies to increase the tolerability of one vs the other, thereby improving quality of life and outcomes through their synergic effects. This expert opinion paper summarizes current data and evolving practices in the management of VHD in patients with HF, including secondary mitral regurgitation, aortic stenosis, aortic regurgitation, and tricuspid regurgitation.

## Graphical Abstract

**Graphical Abstract:**
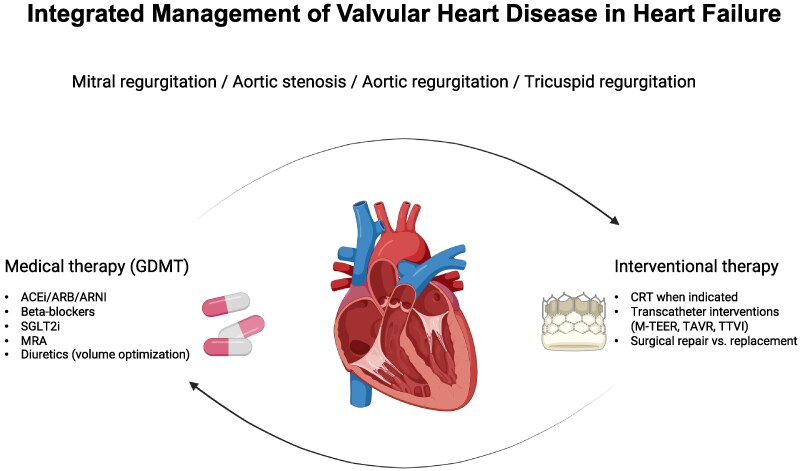
Integrated management of VHD in HF. Traditional management strategies in HF and VHD follow a step-by-step approach, prioritizing GDMT and reserving interventional surgical or percutaneous options only in case of persistent symptoms or worsening HF after GDMT. However, a more integrated approach exploiting the synergistic effects of medical and interventional therapies could be preferred, thus increasing the tolerability of both therapies, eventually improving quality of life and outcomes. Created in BioRender (https://BioRender.com/hv7cv0p). *Abbreviations:* ACEi, angiotensin-converting enzyme inhibitor; ARB, angiotensin-receptor blocker; ARNI, angiotensin receptor-neprilysin inhibitor; CRT, cardiac resynchronization therapy; GDMT, guideline-directed medical therapy; HF, heart failure; MRA, mineralocorticoid receptor antagonist; M-TEER, mitral–transcatheter edge-to-edge repair; SGLT2-I, sodium–glucose co-transporter 2 inhibitor; TAVR, transcatheter aortic-valve replacement; TTVI, transcatheter tricuspid valve intervention; VHD, valvular heart disease.

## Introduction

Heart failure (HF) is a complex and multi-factorial clinical syndrome arising from structural and functional cardiac abnormalities that impair ventricular filling and pump function.^1^ Among the most relevant structural contributors, valvular heart diseases (VHD) are both a cause and a consequence of HF, sharing pathophysiological mechanisms such as neurohormonal activation, chronic inflammation, and adverse cardiac remodelling.^1^ This bidirectional interaction amplifies haemodynamic alterations and accelerates disease progression, making an integrated therapeutic approach essential by targeting multiple pathophysiological mechanisms in parallel. Despite this potential, synergistic approaches remain underutilized in routine clinical practice. Current clinical guidelines tend to promote a stepwise, algorithm-based management: first, maximize pharmacological therapy, and then escalate to surgical or percutaneous interventions, only if symptoms persist. This reflects not only institutional patterns of care, but also the cognitive bias of clinicians towards linear reasoning, even though emerging evidence suggests that earlier, co-ordinated combinations of therapies may improve outcomes.^2–5^ The expansion of percutaneous, minimally invasive techniques has further lowered procedural thresholds, opening the door to hybrid strategies tailored to patient profiles, comorbidities, and disease trajectory.

This expert opinion paper explores the evolving interplay between pharmacological and interventional therapies in patients with VHD and HF, namely secondary mitral regurgitation (MR) (SMR), aortic stenosis (AS), aortic regurgitation (AR), and tricuspid regurgitation (TR) in the context of right HF. Mitral stenosis, whose management is described in the latest guidelines, is not discussed in this article.^6^ We aimed to synthesize current evidence, identify gaps, and propose a framework for integrated care, moving beyond the traditional ‘medical first, intervention later’ mind set towards a more nuanced, patient-centred model that aligns timing, sequencing, and synergy of therapies.

## Secondary mitral regurgitation and heart failure

### Guideline-directed medical therapy optimization before MR correction

**Figure 1:**
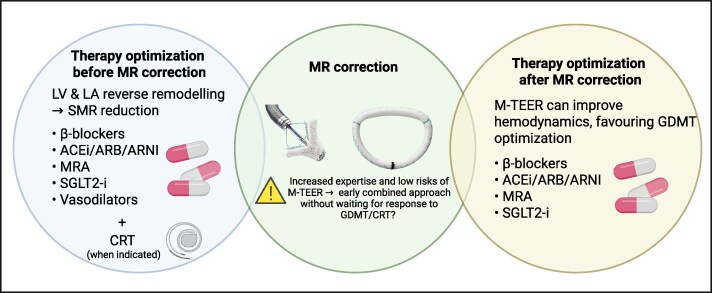
Medical therapy optimization before and after MR correction. The figure shows the necessity of optimize medical therapy (including CRT when indicated) before MR correction due to the possibility of obtaining a MR reduction through LV and LA remodelling. After MR correction, the improvement in haemodynamic favours GDMT optimization, pivotal to maximize MR correction's benefits. Created in BioRender (https://BioRender.com/l1cbytk). ACEi, angiotensin-converting enzyme inhibitor; ARB, angiotensin-receptor blocker; ARNI, angiotensin receptor-neprilysin inhibitor; CRT, cardiac resynchronization therapy; GDMT, guideline-directed medical therapy; LA, left atrial; LV, left ventricular; MR, mitral regurgitation; MRA, mineralocorticoid receptor antagonist; M-TEER, mitral-transcatheter edge-to-edge repair; SGLT2-I, sodium–glucose co-transporter 2 inhibitor; SMR, secondary mitral regurgitation

Optimization of guideline-directed medical therapy (GDMT) represents a pivotal step in the management of SMR, as recommended by current HF guidelines. It is indicated that its implementation and titration should precede consideration of interventional strategies (*Figure 1*).^1^ The rationale for this approach derives from the dynamic nature of SMR, which frequently improves with GDMT through left ventricular (LV) and/or left atrial reverse remodelling with subsequent restoration of mitral valve geometry and improvement in MR severity.^7–9^

Evidence from the first HF studies has already underscored the beneficial effects of neurohormonal antagonists on both LV structure and SMR severity. β-blockade with carvedilol has been shown to favourably impact heart chambers’ geometry, enhance diastolic function and reduce regurgitant volume.^10–12^ Similarly, angiotensin-converting enzyme (ACE) inhibition with captopril has demonstrated reductions in MR severity in patients with dilated cardiomyopathy.^13^ In an analysis of the BIOSTAT-CHF study, up-titration of ACE inhibitors (ACEi) or angiotensin-receptor blockers (ARBs) was associated with MR improvement at 9 months among patients with HF undergoing GDMT optimization.^14^ Moreover, angiotensin receptor-neprilysin inhibitor (ARNI) was associated with significant improvements in MR and LV reverse remodelling.^15,16^ Spinka *et al*. showed that SMR severity improved by at least 1 grade in 39.3% of patients after GDMT titration. Moreover, ARNI as well as the combined dosage effects of (i) renin–angiotensin system inhibitors (RASi) and mineralocorticoid-receptor antagonists (MRA), (ii) β-blockers (BB) and MRA, as well as (iii) RASi, BB, and MRA were all significantly associated with SMR improvement.^17^ Sodium–glucose co-transporter-2 (SGLT2) inhibitors also demonstrated meaningful reductions in MR severity and improvements in myocardial structure in the dapagliflozin effect on functional mitral regurgitation and myocardial remodelling and ertugliflozin for functional mitral regurgitation (EFFORT) trials.^18,19^ Lastly, adjunctive vasodilator therapy with hydralazine has shown outcome benefits in patients with severe systolic dysfunction and concomitant MR when added to conventional regimens.^20^ Recent studies of early implementation of GDMT in patients with new-onset HF with reduced ejection fraction (HFrEF) have shown a reduction of SMR severity in up to 60% of the patients treated.^21,22^ Moreover, compared with no/single GDMT therapy, triple and double therapy before mitral-transcatheter edge-to-edge repair (M-TEER) were independently associated with reduced risk of mortality or HF hospitalization 1 year after intervention.^23^ Therefore, current practice emphasizes the necessity of achieving maximal GDMT titration and allowing sufficient time for reverse remodelling before pursuing transcatheter or surgical correction of SMR. However, it is important to consider that many patients may have medical intolerances, including hypotension and renal failure, which prohibit one or more classes of GDMT and achieving the desired doses.^24^

Taken together, GDMT certainly remains the first-line treatment option in patients with SMR and HF. The impact of the addition of SGLT2 inhibitors, so far missing in most SMR studies, must be evaluated in real-world studies. However, given the high rate of medical intolerances, the increased expertise and low risks of M-TEER, the impact of a combined approach with early percutaneous treatment need to be further evaluated in dedicated trials, as it might further improve the outcomes of these patients without waiting months to evaluate the response to GDMT before considering interventional therapies, and exposing them to a higher risk of adverse events (*Figure 1*).

### GDMT optimization after MR correction

M-TEER would not obviate the need for GDMT; instead, with post-procedural improvement in stroke volume and renal perfusion and increased blood pressure, among the major causes of sub-optimal GDMT titration,^25^ successful M-TEER might allow subsequent GDMT up-titration to maximize procedural benefits and further improve clinical outcomes (*Figure 1*).^7,26^ In EuroSMR, a European multi-centre registry that enrolled 1344 patients with SMR and LV ejection fraction (LVEF) < 50%, triple GDMT prescription (BB, RASi, and MRA) after M-TEER was associated with lower 2-year all-cause mortality compared with non-triple GDMT, particularly in patients with residual SMR ≥2 +.^27^ In a subsequent analysis of the EuroSMR registry, the proportion of patients receiving ACEi/ARB/ARNI, BB and MRA was 78%, 89%, and 62% before M-TEER and 84%, 91%, and 66% at 6 months after M-TEER.^4^ Patients with GDMT up-titration after M-TEER had a lower risk of all-cause mortality and of the composite of all-cause mortality or HF hospitalization compared with patients without up-titration. Moreover, a larger decrease in MR after M-TEER was an independent predictor of increased likelihood of post-procedural GDMT up-titration.^4^ Similarly, Tanaka *et al*. retrospectively analysed 463 patients with LVEF < 50% who underwent M-TEER and observed that triple GDMT (BB, RASi, and MRA) was associated with a lower risk of 2-year mortality as compared with the lack of triple GDMT.^28^ Lastly, observational data from the Japanese multi-centre Optimized Catheter Valvular Intervention (OCEAN)-mitral registry showed that BB up-titration after M-TEER was significantly associated with a lower risk of all-cause and cardiovascular mortality.^29^ In the same registry, low-dose MRA was associated with better clinical outcomes than not administering MRA, though with no further benefit with MRA titration.^30^

However, the rate of GDMT optimization after M-TEER remains sub-optimal. In the analysis by Tanaka *et al*., only 49.2% of patients were treated with GDMT upon discharge.^28^ In the OCEAN-mitral registry, only 18.4% of patients received increasing doses of BB at 1-month follow-up, and most of them received less than the target dose of BB.^29^ In the EuroSMR analysis, 38% of patients underwent GDMT up-titration after M-TEER, but only 3% and 11% of patients received up-titration of three and two drugs, respectively.^4^ Although increased compared with baseline, the proportion of patients on >50% target doses of RASi, BB, and MRA after M-TEER remained low.^4^

Therefore, efforts to further up-titrate GDMT after M-TEER for SMR seem necessary, even if reaching the target dose remains challenging. In the context of HF and SMR, MR correction alone should not be perceived as the final and unique goal, but rather as an opportunity to further intensify GDMT, thus contributing to mitigating HF progression and improving long-term outcomes.

### Complementary interventional procedures: the role of CRT

Cardiac resynchronization therapy (CRT) could play an important role in the management of patients with SMR, when indicated accordingly to current guidelines.^1,31^ In fact, it has been widely demonstrated that CRT is able to reduce SMR severity, as well as induce reverse remodelling. The reduction in SMR, when present, is due to the mechanical effect of CRT, which corrects LV septal wall dyssynchrony. This effect may also be shown acutely, with maximal effects at 3 months and no further improvement thereafter.^32,33^ These effects are known to vary from patient to patient.^2,33–41^ Major predictors of SMR improvement are baseline dyssynchrony, LV size, characteristics of mitral valve apparatus, and LA function.^32,42–50^ However, despite CRT, significant SMR may persist in more than 50% of patients, with a negative impact on outcomes.^35,36,44,51–54^ The current approach in patients with SMR undergoing CRT implantation, on top of GDMT, consists of careful reassessment of SMR severity after a waiting period of at least 3 months.^1^ In case of persistent significant SMR, M-TEER is an important strategy to improve quality of life and prognosis.^7,55–60^ Natanzon *et al*. explored the outcomes of M-TEER in CRT-eligible patients with SMR who did not receive CRT before the procedure, finding that 1-year clinical outcome was more favourable when M-TEER was preceded by CRT.^61^ However, this stepwise approach does not consider the variable probability of response to CRT and the wide availability, by now, of other percutaneous solutions beyond M-TEER, that provide further treatment options for patients with SMR and HF.^62^ Emerging strategies, such as left bundle branch pacing,^63^ and refined phenotyping via multi-modality imaging may further optimize therapeutic choice, indications, and timing.

In summary, CRT represents both potential corrective and prognosis-modifying option in the spectrum of SMR management, although individualized pathways and dynamic SMR reassessment are needed (*Figure 1*).

## Aortic stenosis and heart failure

### Medical therapy and AVR in HFrEF/HFmrEF

**Figure 2:**
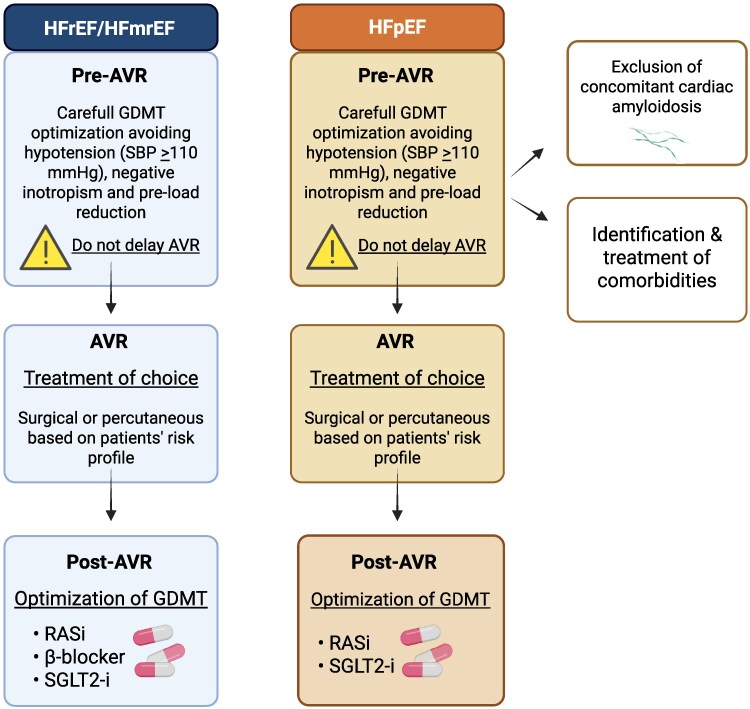
Medical therapy and AVR in patients with HF and severe AS. The figure shows the suggested management in patients with HFrEF/HFmrEF and HFpEF. AVR, through percutaneous or surgical treatment, remains the pivotal step in AS management. GDMT pre-AVR should be administered with caution for the risk of pre-load reduction and hypotension. Post-AVR, GDMT could be a fundamental step to improve outcomes. Created in BioRender (https://BioRender.com/vif19z2). AS, aortic stenosis; AVR, aortic-valve replacement; GDMT, guideline-directed medical therapy; HFmrEF, heart failure with mildly reduced ejection fraction; HFpEF, heart failure with preserved ejection fraction; HFrEF, heart failure with reduced ejection fraction; RASi, renin–angiotensin system inhibitor; RCT, randomized control trial; SBP, systolic blood pressure; SGLT2-I, sodium–glucose co-transporter 2 inhibitor

In patients with severe AS and HFrEF and mildly reduced EF (HFmrEF), surgical or percutaneous aortic-valve replacement (AVR) is the only strategy recommended by the ESC guidelines (*Figure 2*).^1,6^ Evidence from randomized trials supports this approach in patients with LV dysfunction across different risk profiles.^64–72^

Before AVR, optimization of HF medical therapy may be considered while performing diagnostic tests and planning the intervention, but it should be administered with caution and not delay AVR.^73^ Specifically, since AS is a pre-load dependent VHD, drugs that reduce pre-load (e.g. aggressive diuretic strategy) may be detrimental and should be avoided, if possible (*Figure 2*). In this context, a decongestive treatment guided by imaging and not just clinical judgment has been associated with better outcomes and quality of life.^74^ Similarly, hypotensive drugs (including ACEi, ARB, and ARNI) should be used with caution while maintaining a systolic blood pressure target of at least 110 mm Hg. Finally, BB must also be used and titrated with caution because of their negative inotropic effect in the presence of a fixed after-load. SGLT2 inhibitors might be better tolerated than other HF drugs^75,76^ before AVR and could be considered the first-line therapy in these patients, although specific data are lacking.^77^

Considering the limits and risks of pre-AVR GDMT, optimization can be considered mostly after AVR (*Figure 2*). The role of medical treatment post-AVR was investigated in the SWEDEHEART Registry.^78,79^ This analysis showed an association between lower mortality and administration of statins and RASi, but not beta-blockers, with associations that persisted after adjustment for age, sex, and comorbidities. Namely, RASi administration, either ACE inhibitors or ARBs, was associated with a lower risk of major cardiac events, including stroke, myocardial infarction, and mortality, with a major effect on all-cause mortality. Similarly, a multi-centre study explored the potential benefits of RASi on LV remodelling and major clinical outcomes following successful TAVR. Among 2785 included patients, reduction of LV volumes and hypertrophy was greater, and cardiovascular mortality at 3-year follow-up was lower in patients treated with RASi. Moreover, RASi demonstrated a global cardiovascular protective effect with significantly lower rates of new-onset atrial fibrillation (AF), cerebrovascular events and HF readmissions.^80^ However, this study did not specifically analyse patients with LV dysfunction. This data has been partially confirmed in the renin angiotensin system blockade benefits in clinical evolution and ventricular remodelling after transcatheter aortic-valve implantation (RASTAVI) trial, a randomized trial comparing ramipril administration vs usual care in patients with LVEF >40% after successful TAVR. This study did not meet the primary endpoint of cardiac death, HF readmission and stroke at 1 year as compared with placebo, but a significant reduction in HF readmissions at 1 year and LV reverse remodelling without improvement in fibrosis were observed among patients randomized to ramipril.^81^ However, median LVEF was 60% (inter-quartile range 53%–66%) and no subgroup analysis was performed to evaluate the presence of a different response in patients with reduced LVEF. A recent meta-analysis of 13 studies, which also included the results of RASTAVI, confirmed that RASi have benefit on cardiovascular mortality after TAVR as compared with placebo.^82^ Of note, a prospective, single-centre, open-label, randomized trial is ongoing to compare ARNI plus conventional treatment vs. conventional treatment only in patients undergoing TAVR (jRCT1031220344).^83^

In contrast to the benefits shown by RASi and SGLT2 inhibitors, treatment with BB was not associated with long-term outcome after SAVR in a previous analysis of the SWEDEHEART Registry, but this study was not specifically focused on patients with LV dysfunction.^84^ In an analysis of the OCEAN-TAVI Registry, BB use was associated with lower 2-year cardiovascular mortality among patients with LVEF after TAVR <50%.^85^

In the dapagliflozin in patients undergoing transcatheter aortic-valve implantation (DAPA-TAVI) trial, 1222 patients with HF and at least one risk factor between diabetes, chronic kidney disease and LV dysfunction, were randomly assigned to receive either dapagliflozin or standard of care. At 1-year follow-up, the risk of the primary composite endpoint of death from any cause or worsening HF events was reduced by 28% in the treatment arm as compared with the control arm, mainly driven by worsening HF event.^86^ It must be, however, noted that the mean LVEF was 55% ± 12% with only 16%–18% of the patients with a LVEF <40%. No heterogeneity was noted, however, when the patients were sub-divided based on their LVEF. Consistently, in a multi-centre international registry of 311 consecutive diabetic patients with severe AS and LVEF <50% undergoing TAVR, use of SGLT2 inhibitors was independently associated with reduced all-cause mortality and HF hospitalizations.^87^ In addition, patients treated with SGLT2 inhibitors experienced a higher rate of LV recovery, especially those with baseline LVEF ≤30%.

In summary, in the presence of severe AS and HFrEF or HFmrEF, SAVR, or TAVR represents an effective disease-modifying treatment, but efforts to optimize concomitant GDMT, namely RASi and SGLT2 inhibitors, seem warranted. Before AVR, GDMT may contribute to stabilization and optimization of haemodynamics, but must be used with caution. After successful AVR, the implementation of GDMT may have a major role to consolidate reverse remodelling, prevent recurrent HF events, and improve long-term outcomes, including all-cause mortality. Future studies should address the optimal integration and sequencing of GDMT with AVR to maximize patient benefits.

### Medical therapy and AVR in HFpEF

Patients with AS often have HFpEF. HFpEF can be entirely secondary to AS (concentric hypertrophy/remodelling), multi-factorial (AS plus other conditions, such as amyloidosis) or primarily unrelated to AS.^88–90^ It may therefore have a different outcome from patient to patient after TAVR.^90^ AS and HFpEF share key haemodynamic features, a small and rigid left ventricle, elevated ventricular and arterial elastance, pre-load dependence, and after-load sensitivity. Increased aortic stiffness is a hallmark of AS,^90–92^ but may also be found in HFpEF.^90,93^ After TAVR, patients with higher arterial stiffness may show limited reverse remodelling.^94^ Therefore, in severe AS with preserved LVEF, integrating HFpEF scoring systems may refine prognostic stratification pre-TAVR.^95–97^ Reassessment of HF after AVR is crucial to clarify these mechanisms and tailor treatments.

Before AVR, similarly to HFrEF/HFmrEF, medical therapy optimization should be considered during diagnostic work-up, also in patients with AS and HFpEF, without delaying AVR when indicated (*Figure 2*). Congestion is common in both AS and HFpEF, as evidenced by clinical signs, B-lines, dilated inferior vena cava and renal flow abnormalities.^90,98^ Careful diuretic titration is needed to relieve symptoms while avoiding excessive pre-load reduction and hypotension. SGLT2 inhibitors, currently the only guideline-recommended medical treatment for HFpEF, appear well tolerated in AS, may reduce congestion and, potentially, slow AS progression,^99^ although randomized data on the latter effect are lacking. Their ability to modulate interstitial fluid balance, preventing dangerous pre-load decrease, makes them particularly attractive before AVR. Treatment of comorbidities should be a priority both pre- and post-AVR. Proper management of hypertension is critical, since elevated after-load accelerates LV hypertrophy and worsens prognosis.^100,101^ RASi are a first-line treatment, with evidence of positive LV remodelling in AS,^102,103^ but with a risk of hypotension.^104,105^ Data on MRAs are limited in this setting: a small trial on eplerenone was neutral,^106^ but pathophysiological rationale supports potential benefit in patients with myocardial fibrosis and diastolic dysfunction.^107,108^ Metabolic comorbidities, such as obesity, accelerate both AS and HFpEF progression^109,110^ and its treatment may enhance post-AVR recovery.

After AVR, tailored HFpEF therapies^111^ should be considered for persistent symptoms or management of comorbidities. In patients with LVEF >40%, DAPA-TAVI showed benefits on death from any cause or worsening HF events,^86^ while RASTAVI showed a significant reduction in HF readmissions at 1 year.^81^

In summary, AS and HFpEF share pathophysiology and clinical features, yet therapies validated for HFpEF remain scarcely tested in AS. Optimization of pre-load and after-load management before AVR, and aggressive treatment of comorbidities after AVR, are crucial in this setting. Future research should address the role of new therapies that have recently demonstrated benefits in HFpEF, such as finerenone and semaglutide, also in the specific subset of patients with AS and HFpEF.^112–116^ These treatments are currently unstudied in this setting but have a strong pathophysiologic rationale in terms of fibrosis modulation, metabolic effects, and weight reduction.

### Moderate aortic stenosis

**Figure 3:**
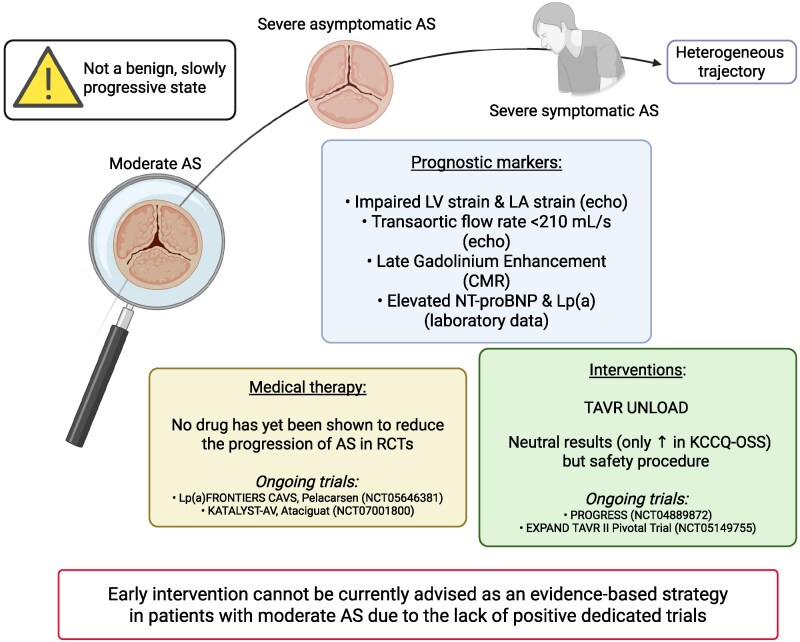
Moderate AS. The figure shows the heterogeneous trajectory of moderate AS. One time considered a benign, slowly progressive state, now it is considered associated with poor outcomes. No drug has yet been shown to reduce the progression of AS in RCTs. TAVR UNLOAD, a RCT, did not shown strong benefit although the procedure was safe. Created in BioRender (https://BioRender.com/40o8ze1). AS, aortic stenosis; CMR, cardiac magnetic resonance; KCCQ-OSS, Kansas city cardiomyopathy questionary–overall summary score; LA, left atrial; LV, left ventricular; RCTs, randomized control trials

Moderate AS has traditionally been considered a benign, slowly progressive state (*Figure 3*). However, accumulating evidence now highlights its substantial prognostic impact in patients with HF,^117^ especially when LV dysfunction is present.^73^ Moderate AS may exacerbate LV remodelling, increase after-load and accelerate clinical deterioration, challenging long-standing perceptions of this condition as a passive or inconsequential stage.^118–126^ Importantly, moderate AS is a dynamic condition with a heterogeneous trajectory.^127^ Some patients advance rapidly to severe AS, emphasizing the need for early identification and risk stratification.^128^ Prognostically relevant imaging markers, including late gadolinium enhancement on cardiac magnetic resonance, impaired LV global longitudinal strain, impaired left atrial strain or reduced transaortic flow rate (<210 ml/s) can reflect myocardial fibrosis and sub-clinical dysfunction, even in patients with preserved LVEF.^117,129–133^ Serial echocardiographic follow-up, ideally augmented with artificial intelligence-based prediction models, is therefore critical for timely intervention.^134^ Beyond imaging, biomarkers such as N-terminal pro–B-type natriuretic peptide (NT-proBNP), a surrogate of LV wall stress, have demonstrated prognostic value. In a cohort of 261 patients with moderate AS, elevated NT-proBNP levels were independently associated with higher mortality.^135^

Lipids may play a role in AS progression. Sub-studies from the aortic stenosis progression observation: measuring effects of rosuvastatin study highlighted that elevated lipoprotein(a) were associated with faster AS progression and bioprosthetic valve deterioration.^136–138^ However, in the simvastatin and ezetimibe in aortic stenosis trial, intensive lipid lowering therapy with simvastatin and ezetimibe did not significantly alter AS progression or major cardiovascular events.^139^ This data was confirmed in a meta-analysis of four randomized trial enrolling 2344 patients.^140^ An ongoing trial is testing anti-lipoprotein(a) therapy in early stage AS (Lp(a) FRONTIERS CAVS, NCT05646381). Interestingly, recent data suggest a potential disease-modifying effect from SGLT2 inhibitors.^99^ A target trial emulation using electronic medical records showed that patients with moderate AS treated with SGLT2 inhibitors had a lower risk of progressing to severe AS, with an incremental benefit related to treatment duration (hazard ratio of 0.54, 0.48, and 0.27 with SGLT2 inhibitors for >3, 6 and 12 months, respectively), although this promising data needs to be confirmed in proper dedicated studies. Reactivation of oxidized soluble guanylate cyclase, the primary receptor for nitric oxide, could also be an efficacious strategy to slow AS progression. The Phase 3, randomized, study checking the efficacy and safety of ataciguat to slow the progression of valvular dysfunction in participants with moderate calcific aortic-valve stenosis (KATALYST-AV and NCT07001800) randomized trial is ongoing to test this hypothesis.

The yield of intervention in moderate AS has become a topic of great interest and active investigation. Although the transcatheter aortic-valve replacement to unload the left ventricle in patients with advanced heart failure (TAVR UNLOAD) randomized trial^141^ did not meet its primary composite endpoint, it demonstrated a clear safety profile for TAVR and significant improvements in quality of life among patients with moderate AS and LV dysfunction. Despite several challenges and limitations^142^ TAVR UNLOAD reinforced the concept that pre-emptive valve intervention might be beneficial in selected patients with moderate AS and HF, particularly in terms of symptom relief and quality of life. A recent propensity score-matched analysis confirmed the benefits of early TAVR in moderate AS.^143^ Of note, the prospective, randomized, controlled trial to assess the management of moderate aortic stenosis by clinical surveillance or transcatheter aortic-valve replacement (NCT04889872) and the Evolut™ EXPAND TAVR II pivotal trial (NCT05149755) studies are currently recruiting patients to explore the hypothesis that TAVR could improve outcomes in patients with moderate AS who have symptoms or evidence of cardiac damage or dysfunction.

In summary, moderate AS is increasingly recognized as a prognostically relevant condition in HF, especially among patients with reduced LVEF. However, no randomized trial has yet demonstrated a benefit on hard clinical outcomes. Therefore, early intervention cannot currently be recommended as an evidence-based strategy. Ongoing trials will clarify the role and timing of AVR in this population.

## Aortic regurgitation and heart failure

In patients with severe AR and HF, SAVR, aortic-valve repair or TAVR are the interventional options recommended by the latest ESC guidelines.^1,6^ Severe AR can lead to progressive LV dilation with subsequent dysfunction and HF, leading to poor prognosis. Focused clinical data on medical therapy in this setting are scarce, and treatment should be regarded mainly as a bridge to intervention. Pre-procedurally, diuretics may alleviate symptoms of congestion, while vasodilators such as ACEi or sacubitril/valsartan can reduce after-load and regurgitant volume. In an observational study, prescription of ACEi or ARBs in patients with moderate to severe AR was associated with significantly reduced all-cause mortality and cardiovascular and AR-related events.^144^ Calcium antagonists were initially shown as potentially useful in patients with AR.^145^ However, a subsequent trial failed to show any effect of long-term vasodilator therapy with nifedipine or enalapril on the need for AVR in patients with asymptomatic severe AR and normal LV systolic function.^146^ These agents may therefore be useful to treat concomitant conditions that may worsen AR (i.e. hypertension) but are not indicated to delay the progression of AR. Evidence on SGLT2 inhibitors is lacking in the specific AR setting, yet their safety and efficacy in other HF and VHD populations suggest they may be a reasonable option even before AVR. Conversely, BBs require caution, since by prolonging diastolic time, they can increase regurgitant volume; they may be considered only in selected patients with LV dysfunction, avoiding bradycardia. Despite this, an observational study including 756 patients with severe AR showed that BB therapy was associated with improved survival compared with those not receiving it, even after adjustment for LVEF, comorbidities, and valve intervention, suggesting that potential benefits may outweigh risks.^147^ After successful AVR, GDMT becomes central to long-term management, as in other VHD, with the potential to enhance LV reverse remodelling and improve clinical outcomes and beta-blockers can be used along with ACE inhibitors or ARBs to treat LV systolic dysfunction.^6^

In summary, in severe AR with HF, medical therapy has only a supportive role as a bridge to intervention, with diuretics and vasodilators for stabilization. GDMT should be optimized after AVR or aortic-valve repair to promote reverse remodelling and improve outcomes.

### Aortic regurgitation in LVAD

**Figure 4:**
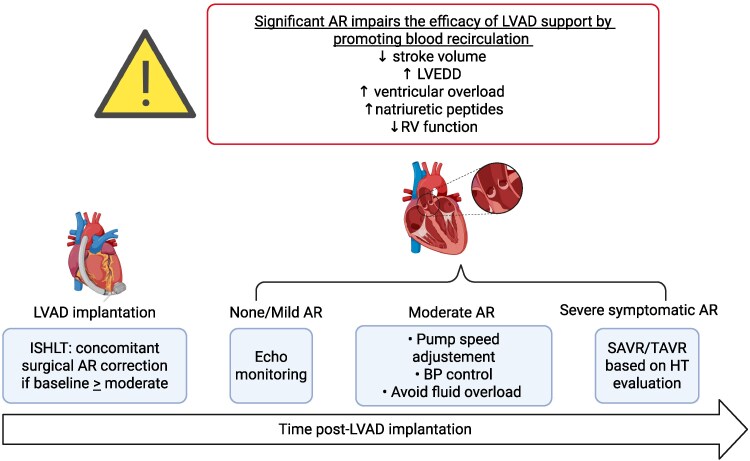
Aortic regurgitation in patients with LVAD. The figure shows the management of AR in patients with LVAD. Created in BioRender (https://BioRender.com/yc7ghm6). AR, aortic regurgitation; BP, blood pressure; ISHLT, international society for heart and lung transplantation; LVAD, left ventricular assist device; LVEDD, left ventricular end-diastolic diameter; RV, right ventricular; SAVR, surgical aortic-valve replacement; TAVR, transcatheter aortic-valve replacement

AR is common and clinically relevant in patients supported with durable continuous-flow LVADs (*Figure 4*). Its incidence varies according to device type and support duration, affecting up to 37% of patients post-LVAD implantation.^148–150^ Moreover, the diagnosis and grading of AR in LVAD patients remains challenging due to non-standardized assessment criteria.^151^ The pathophysiology of AR in LVAD patients is multi-factorial. Persistent valve closure and altered transvalvular flow dynamics lead to commissural fusion, cusp remodelling and structural changes in the aortic root. These changes are exacerbated by excessive LV unloading and abnormal biomechanics at the outflow graft–aortic anastomosis. Risk factors include older age, female sex, smaller body size, arterial hypertension, large aortic root diameter, permanently closed aortic valve, longer support duration, and unfavourable graft angle.^152^

The development of significant AR impairs the efficacy of LVAD support by promoting blood recirculation. This determines a reduction in the stroke volume and an increase in the LV end-diastolic diameter with consequent LV overload and an increase in natriuretic peptides. In addition, there may also be impairment of RV function. From a clinical point of view, all this manifests itself with recurrent symptoms of HF, in addition to the possible presence of extra-cardiac symptoms such as an increased tendency to gastrointestinal bleeding due to increased mucosal fragility.^153–155^

Prognostic data are controversial. Early studies^156,157^ showed no significant difference in survival between patients who developed moderate/severe AR and those who did not, particularly with HeartMate II devices. However, more recent evidence with HeartMate 3 LVAD indicates that progressive AR is associated with increased rates of HF rehospitalization and all-cause mortality.^158^ INTERMACS registry data^155^ support these findings, showing higher 2-year mortality and rehospitalization rate in patients with at least moderate to severe AR. Given the progressive nature and haemodynamic burden of AR, which compromises the function of the LVAD itself, pro-active intervention is increasingly favored.^159^ The 2023 International Society for Heart and Lung Transplantation (ISHLT) guidelines^160^ recommend surgical correction of AR at the time of LVAD implantation in patients with more than mild-AR (class I, level C). However, data on concomitant aortic-valve procedures are conflicting,^161^ with some evidence suggesting increased early and late mortality in the AVR group,^162,163^ and with differences varying by the type of intervention.^164^ In cases where concomitant surgery on the aortic-valve is not performed, it is important to monitor the patient to assess the development of significant AR by serial echocardiographic follow-up. In patients who develop significant AR, management strategies include pump speed optimization, careful treatment of arterial hypertension and volume overload.^154^ If haemodynamic compromise persists, valve intervention becomes necessary. TAVR has emerged as a viable option, especially for high-risk or inoperable patients.^165–170^ A retrospective analysis by Zaidi *et al*. comparing TAVR vs. SAVR in patients with prior LVAD support found that TAVR was associated with shorter hospital stays, fewer complications and comparable 30-day mortality.^171^

In summary, AR is a frequent, progressive complication in LVAD patients that impairs device efficacy and worsens outcomes, particularly with newer-generation devices. Future research should focus on standardized diagnostic criteria, refining timing and type of interventions (including TAVR), and assessing long-term outcomes to optimize management strategies.

## Tricuspid regurgitation and right HF

### Medical therapy and phenotyping before TR correction

**Figure 5:**
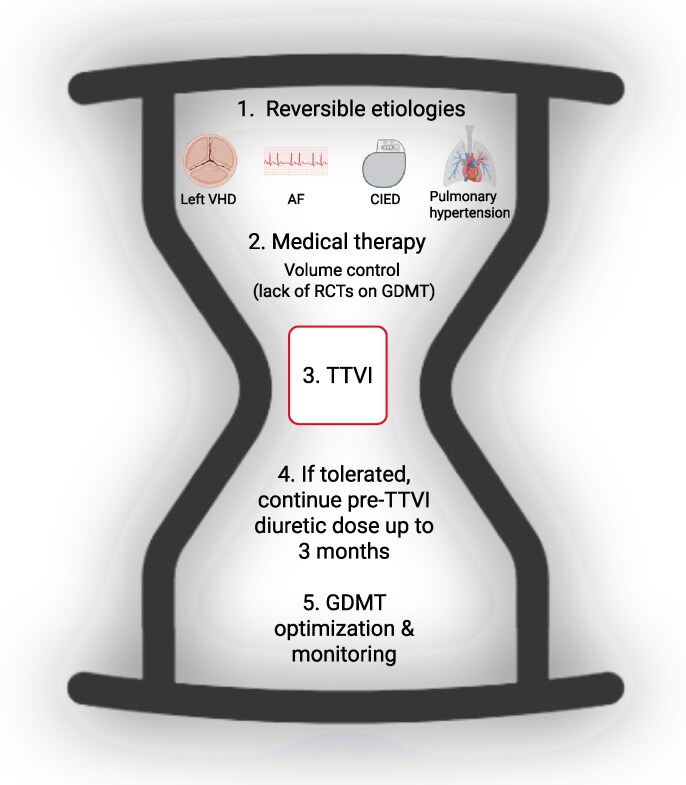
Management of patients before and after TTVI. The figure shows the suggested management in patients with severe TR and right HF. The first step should be to identified as reversible TR aetiologies, including left VHD, AF, CIED-related TR and pulmonary hypertension. In patients without these aetiologies or after the management of these aetiologies, persistency of TR should be managed with medical therapy, particularly with diuretics to avoid fluid overload without lingering on TTVI, if indicated. After TTVI, diuretics should ideally be continued up to 3 months, if tolerated, to avoid device detachment. GDMT optimization post-TTVI could be useful to improve outcomes, but data are lacking. Created in BioRender (https://BioRender.com/xh2xt8j). AF, atrial fibrillation; CIED, cardiac implantable electronic device; GDMT, guideline-directed medical therapy; RCTs, randomized control trials; TTVI, transcatheter tricuspid valve intervention; VHD, valvular heart disease

Management of right HF and TR should begin with the identification and treatment of potentially reversible aetiologies (*Figure 5*).^172^ This includes assessment of left-sided valvular disease, AF, pulmonary hypertension (PH), and cardiac implantable electronic device (CIED)-related TR, as these conditions strongly influence treatment strategy and prognosis.^173^

In patients with left-sided valve disease, concomitant tricuspid valve intervention during mitral or aortic surgery is recommended in case of severe TR, but it also should be considered when moderate TR is present and in case of annular dilation.^6^ For high-risk surgical candidates, staged transcatheter tricuspid valve intervention (TTVI) approaches, either repair or replacement, may be considered. Improvement of TR after left-sided transcatheter procedures is reported,^174,175^ yet persistent moderate-to-severe TR confers adverse prognosis and warrants reassessment for dedicated TR intervention.^176,177^

Management of AF-related TR focuses on rhythm control, when arrhythmia is the presumed direct cause and not a consequence. Rhythm control strategies achieving durable sinus rhythm, including catheter ablation, may reduce annular dilation and improve TR severity.^178–182^ However, lack of TR improvement 6 months after catheter ablation is associated with an increased risk of AF recurrences.^183^ Similarly, PH should be managed with disease-specific therapies according to the current PH guidelines.^184^ Patients with CIED-related TR should be managed by a multi-disciplinary team.^185,186^

Early transvenous lead extraction may be considered when mechanical leaflet interference is demonstrated, but it carries a risk of worsening regurgitation. If extraction is not feasible and the patient is eligible for TTVI, the risk of lead entrapment by replacement devices must be balanced against alternative pacing strategies or non-entrapping device therapies.

When no reversible aetiology is identified or when TR persists despite targeted therapy, medical management is primarily focused on volume control.^1^ Loop diuretics remain the mainstay for relieving systemic congestion and avoid recurrent hospitalization.^187^ Combination diuretic therapy or escalation to inotropes/vasopressors may be necessary in acute and refractory cases, particularly when end-organ hypoperfusion is evident.^188–190^ Unlike left-sided HF, no neurohormonal modulator has demonstrated prognostic benefit in randomized trials on right HF. However, observational studies suggest potential improvements in RV function with sacubitril/valsartan,^191–194^ MRAs^195,196^ and SGLT2 inhibitors.^197–199^ Importantly, in view of the absence of randomized data, optimization of volume status should not delay evaluation for TTVI when clinically indicated. Reassessment with comprehensive echocardiography and right heart catheterization under euvolemic conditions is recommended before finalizing procedural candidacy. This step is critical to distinguish reversible functional TR from advanced RV involvement, which may predict poorer response to intervention as well as clinically defined severe right HF.^172,200–202^

In summary, systematic phenotyping and targeted treatment of reversible causes remain crucial to optimizing outcomes before tricuspid valve interventions. Future studies should clarify the impact of early RV reverse remodelling and neurohormonal therapies on procedural success and long-term survival and define standardized algorithms for timing of interventions and patient selection.

### Medical therapy after TR correction

Following TTVI, residual TR is relatively common and associated with worse outcomes, particularly in the presence of PH.^203–206^ These factors, along with pre- and post-procedural RV function, may influence the degree of decongestion required in the early post-procedural phase. An increase in urine output within the first 24–48 h after TTVI has been observed, likely related to decreased central venous pressure and improved renal perfusion gradients.^207,208^ However, if tolerated by the patient, pre-procedural diuretic dosage should be continued at least up to 3 months after TTVI with constant body weight (under continuous daily weight monitoring) (*Figure 5*).^209^ This may prevent early fluid overload and promote reverse RV remodelling.^210^

In the trial to evaluate cardiovascular outcomes in patients treated with the tricuspid valve repair system (TRILUMINATE) study, loop diuretic doses remained largely unchanged at discharge and only gradually declined at 6 months; unexpectedly, 7.5% of patients required dose escalation or initiation of new diuretic therapy after TTVI.^211^ Moreover, renal function frequently remains stable or improves following correction of severe TR.^212–214^ Mild improvements in hepatic function have also been reported. These favourable changes in end-organ function may enable up-titration of GDMT that are often limited in advanced right-sided HF. Despite this potential, data regarding GDMT changes after TTVI are poor.^215^

In the TRILUMINATE trial, use of ACEi was low (<20%), and dose adjustments were infrequent (only in 12% of patients), while in 24% of patients the dose was decreased^211^; BB were prescribed in over 65% of patients enrolled in TRILUMINATE and largely maintained.^211^ No data are available about MRA and SGLT2 inhibitors changes after TTVI, although these agents may confer additional benefits, particularly in terms of volume overload and long-term outcomes.

In summary, TTVI can lead to early improvements in renal and hepatic function, potentially facilitating GDMT optimization. However, available data on post-procedural GDMT adjustments remain scarce, with most trials reporting minimal changes in neurohormonal blockade or diuretic use. Future studies should systematically capture these modifications to clarify their impact on clinical outcomes.

## Conclusions

HF and VHD often coexist, driving disease progression through shared pathophysiological mechanisms. Emerging evidence could support earlier and more integrated use of interventional strategies alongside optimized medical therapy, facilitated by advances in percutaneous techniques. While this approach might improve symptoms and functional status, robust randomized data remain limited, particularly for early intervention in moderate AS, early M-TEER in SMR, and structured GDMT optimization after valve interventions. A patient-centred, multi-disciplinary model that aligns the timing and synergy of therapies is essential to maximize outcomes.

## Author contributions

All authors contributed to conceptualization, article writing, and article review/editing.

## Declarations

### Disclosure of Interest

M.P. has received personal fees from Abbott Vascular, AstraZeneca, Bayer, Boehringer Ingelheim, Eli Lilly, Novartis, Novo Nordisk, Roche Diagnostics, Viatris, and Vifor Pharma. F.M. has received grant and/or research institutional support from Abbott, Medtronic, Edwards Lifesciences, Biotronik, Boston Scientific Corporation, NVT, Terumo, Venus and Roche; consulting fees, personal and institutional honoraria from Abbott, Boston Scientific, Medtronic, Edwards Lifesciences, Xeltis, Cardiovalve, Occlufit, Simulands, Mtex, Venus, Squadra, Valgen, CroiValve, Meril, and Balmed; royalty income/IP rights from Edwards Lifesciences; and is shareholder (including share options) of Magenta, Transseptalsolutions, and 4Tech. M.Ab. has received speaker honoraria from AstraZeneca, Bayer, Boehringer Ingelheim and Novartis. E-L.A. has received honoraria for lectures from AstraZeneca, Berlin Chemie, Boehringer Ingelheim, Ewo, Novartis, Servier and Vifor Pharma. N.K. has received consultant fees from Edwards Lifesciences, Boston Scientific and Medtronic; and proctor fees from Abbott. D.M. has received speaker honoraria from GE Healthcare, Philips, Edwards, and Bristol-Myers Squibb. M.Ad. has received consulting fees in the last 3 years from Abbott Structural Heart and Edwards Lifesciences. M.M. has received consulting fees in the last 3 years from AstraZeneca, Bayer, Boehringer Ingelheim, NovoNordisk and Roche Diagnostics. All other authors have reported that they have no relationships relevant to the contents of this paper to disclose.

## Data Availability

No data were generated or analysed for this manuscript.

## Pre-registered Clinical Trial Number

None supplied.
